# The Developmental Processes of *Dolycoris baccarum* (L.) (Hemiptera: Pentatomidae) Under Different Temperature Regimes

**DOI:** 10.3390/insects16121245

**Published:** 2025-12-09

**Authors:** Rameswor Maharjan, Seo Yeon Hong, Jun Hyoung Jeon, Jeong Joon Ahn, Young Nam Yoon, Ok Jae Won, Hyeon Su Lee, Jee-Yeon Ko

**Affiliations:** 1 Smart Agricultural Technology Division, Department of Upland Crop Research & Development, National Institute of Crop Science, Rural Development Administration, Miryang 50424, Republic of Korea; mrameswor@gmail.com (R.M.); yoonyn@korea.kr (Y.N.Y.); ojwon@korea.kr (O.J.W.); ihyeonsu773@korea.kr (H.S.L.); kjeeyeon@korea.kr (J.-Y.K.); 2 Research Institute of Climate Change and Agriculture, National Institute of Crop Science, Rural Development Administration, Jeju 63240, Republic of Korea; j2ahn33@korea.kr

**Keywords:** shield bug, models, spring emergence, forecasting, population dynamics, management

## Abstract

Understanding how insects adapt to temperature is crucial to elucidating the ecological factors shaping their life history traits. Phenological models are influenced by temperature, allowing researchers to examine how temperature affects population dynamics, geographical distribution, and the management of various insect species. This study was conducted at seven constant temperatures (15.3, 20.8, 25.0, 27.0, 30.1, 35.0, and 40.0 °C) under temperature-controlled conditions in an incubator to assess temperature-dependent development of *D. baccarum*. Clusters of eggs were put into Petri dishes and kept in a humidity chamber. The humidity chamber was then placed inside the incubator. Temperature affected the developmental parameters and mortality of *D. baccarum* reared on sesame seed pods. Stage-specific parameters, including the lower developmental threshold (LDT) and thermal constant (K, in degree days [DD]), were estimated using linear (GLM) and nonlinear (Lactin2) models, respectively. Total development time from egg to adult decreased with increasing temperature. Successful development occurred between 20.8 and 35.0 °C, and failed under 15.3 and 40.0 °C (100% nymph mortality). Egg stage duration ranged from 30.56 days at 15.3 °C to 2.07 days at 40 °C, while nymphal development ranged from 64.75 days at 20.8 °C to 21.17 days at 35.0 °C. The estimated LDT and K-values were 14.22 °C and 492.22 degree days (DD), respectively. Based on these thermal requirements, we developed a predictive model to better understand population dynamics and inform pest management strategies, which can help predict the spring occurrence, number of generations, and population dynamics of *D. baccarum*.

## Simple Summary

The shield bug *Dolycoris baccarum* (Hemiptera: Pentatomidae) poses a significant threat to the cultivation of various plants in Korea. Prompt management is crucial since delays can result in significant economic losses. The creation of accurate forecasting models is vital for effective pest management. Therefore, we assessed the temperature-dependent thermal requirements of *D. baccarum* using both linear and nonlinear models from data of sesame seed pods to create models that can predict its spring emergence and population trends. Temperature had a considerable influence on the growth of *D. baccarum*. Development slowed as temperature became less optimal, with no development at very high or low temperatures. Utilizing the estimated thermal requirements, we developed a model predicting the spring emergence of *D. baccarum* in June and July. Continuous monitoring in agricultural fields is necessary to develop better management strategies. This is the first study that clarifies the developmental processes of a Korean population of *D. baccarum* infesting sesame fields.

## 1. Introduction

Pentatomids and alydid hemipterans pose significant economic threats to leguminous, cereal, and fruit crops in Korea [1,2]. They inflict greater harm on soybean (*Glycine max* L.) (Fabaceae) and sesame (*Sesamum indicum* L.) (Pedaliaceae) than on other crops. Both mature adult bugs and nymphs damage the seeds of different leguminous crops and fruits by puncturing and extracting milky and oily substances and fruit juice, ultimately reducing seed quality, germination capacity, and yield [1,3,4,5]. Moreover, they damage crops and fruits and transmit viral, bacterial, and fungal diseases [6]. In Korea, six species of hemipterans, *Riptortus pedestris* Fabricius, *Piezodorus hybneri* Gmelin, *Nezara antennata* Scott, *Dolycoris baccarum* L., *Halyomorpha halys* Stål, and *Plautia stali* Scott (Hemiptera: Pentatomidae), are major stink bug pests known to attack field crops and fruits [7]. Among hemipterans, *D. baccarum* is commonly found across Asian and European continents [8], where it feeds on various plant species and damages several crop species [9,10,11]. *Dolycoris baccarum* overwinters in adult diapause [10,12], and the number of generations it produces is dependent on the temperature and photoperiod conditions of its geographic location [13].

The impact of the climate on organisms, communities, and the broader environment is a serious concern for biologists and environmental scientists. Recent research has shown that earlier estimates were conservative regarding the extent of global warming [14]. Current climatic models predict that the global average surface temperature will rise by 1.8 to 4 °C by the year 2100 [15]. The ecological consequences of climate change driven by global warming are evident in observed effects on species fitness [16,17], range shifts [18], species interactions [19], and community structure [20]. As ectothermic organisms, insects are physiologically reactive to external environmental conditions. Temperature, in particular, plays a critical role in determining their developmental rate, biological traits such as sex ratio, adult lifespan, survival rates, and egg laying, adult size, viability in insect species, and geographical distribution [21,22,23,24,25,26,27]. Several previous studies have demonstrated that insects develop rapidly at higher temperatures; however, adult size varies under extreme temperature regimes [28,29]. Several hypotheses have been proposed to explain this developmental phenomenon in poikilothermic organisms. However, a universally accepted, fact-based common law applicable to all such organisms has yet to be recognized [13,28]. Pest forecasting, management, and risk assessment rely on obtaining information regarding the growth, survival, and reproduction of pests under different environmental conditions. Insect growth is highly responsive to temperature fluctuations, and minor shifts can result in significant disparities in insect phenology [30,31,32]. Forecasting the seasonal presence and populations of insect pests is vital for developing effective and sustainable management strategies. These predictions necessitate comprehending the connection between insect development rate and temperature, particularly concerning potential climate change [33,34], which is frequently represented in temperature-driven phenology models utilizing degree-days [35].

In Europe, detailed studies on the life cycle adaptation of *D. baccarum* have been conducted [36,37,38,39,40]. There have been a few studies on the photoperiod and temperature-induced development of *D. baccarum* in China [41] and Japan [10,13]. Numerous studies on temperature dependence have been conducted on several pentatomids [42,43,44]. However, studies examining the effects of constant temperature on the development of *D. baccarum* on sesame crops in Korea are lacking, with the exception of trap monitoring, occurrence [7,45,46], and biocontrol studies [47,48]. This study investigated the effects of constant temperature on the developmental processes and estimated thermal requirements of *D. baccarum* on sesame (*Sesamum indicum* L.) pods. Additionally, this study created predictive timing for pest emergence by assessing the thermal needs of *D. baccarum* within the context of climate change to ensure the sustainable management of hemipteran bugs, including *D. baccarum*.

## 2. Materials and Methods

### 2.1. Insect Collection and Rearing

Adult *D. baccarum* were collected from a sesame field in the Department of Upland Crop Research & Development, National Institute of Crop Science, Rural Development Administration (35°49′40″ N, 128°74′01″ E), Miryang, Gyeongnam Province, Korea, in 2023. The collected adults were reared on green sesame pods attached to plant stems inside acrylic cages (40 × 40 × 40 cm, with side ventilation). Additionally, dried sesame seeds and water were also provided in a Petri dish with wetted cotton as food and water sources, respectively. After the eggs were laid, they were collected and placed in a Petri dish for nymph hatching. Bugs were reared under laboratory conditions (26 ± 1 °C, 60 ± 5% relative humidity (RH), and a 12:12 h L:D photoperiod). For the experiment, egg clusters attached to sesame pods and stems were collected by clipping the pods and stems, and these clusters were used in the experiment.

### 2.2. Sesame Plant

Sesame plant (cv. Milsung) [49] seeds were sown in plastic pots (18 cm in diameter × 13 cm H) filled with commercial soil media (Baroker, Seoul Bio., Seoul, Republic of Korea), without chemical fertilizers and pesticides. Plants were grown in a greenhouse at 28 ± 2 °C and 60–80% RH under natural photoperiod conditions. Plants were raised until seed pods formed, which were used to rear the bugs.

### 2.3. Experimental Procedure

The study commenced by introducing adult bugs to sesame plants (podding stage) grown in a greenhouse for 3–4 days so we could collect eggs. Exposure occurred in acrylic cages (40 × 40 × 40 cm) with lateral ventilation, maintained at 26 ± 1 °C, 60 ± 5% RH, and with a 12:12 h L:D photoperiod cycle. During this period, adult bugs were provided with dry sesame seeds and water. For the experiment, eggs were collected from plants 2–3 days after exposure to adult bugs. After oviposition on sesame seed pods/stems/leaves, 1 or 2 egg clusters (nonoverlapping) were isolated and counted for each observation. Egg viability was assessed by examining the shape, size, and color of the eggs under a microscope (LEICA EZ4D, Wetzlar, Germany) [50]. Clusters of eggs fixed to sesame seed pods/stems/leaves were transferred to a Petri dish (9.8 cm dia. × 4 cm H) without food sources and stored in a humidity chamber (27 × 20 × 17 cm), maintaining 70–75% RH using saturated salt (NaCl) solutions (Duksan Pure Chemicals, Ansan-si, Republic of Korea), as outlined in [51], for a short period. Before placing the humidity chamber in the incubator, each humidity chamber was equipped with a HOBO data logger (Huato Log-USB; Huato Electronic Co., Ltd., Baoan District, Shenzhen, China) to record the actual temperature and humidity levels within the incubators. Afterward, the humidity chambers were moved to the incubators (Eyela, model MTI-202B, Tokyo, Japan), where the temperatures were adjusted to 15.3, 20.8, 25.0, 27.0, 30.1, 35.0, and 40.0 °C, with a 12:12 h L:D photoperiod. Hatching duration under the various temperatures was recorded. Each freshly emerged individual nymph was placed in individual Petri dishes (5 cm dia. × 1.5 cm H, featuring topside ventilation) containing a single green sesame pod. Individual Petri dishes containing one nymph per seed were sealed with parafilm (PM-996, Amcor Flexibles, 101 Oakley Street, Evansville, IN, USA) and maintained in the same humidity and incubators. The single green pod in each Petri dish was replaced every 2–3 days, with the dish resealed using parafilm. The duration of each developmental phase (egg, nymph, and adult) at each temperature was observed every 24 h until the emergence of adult bugs or death. Each adult bug was placed in a Petri dish (5 cm dia. × 1.5 cm H, with ventilation on top) to assess adult bug lifespan at the corresponding temperatures. The adult bugs received green sesame pods as feed.

### 2.4. Developmental Distribution Model

The Weibull function is a continuous probability distribution that can model various shapes. It can be utilized in numerous data analyses and forecasting tasks with limited sample sizes. The cumulative Weibull function describes the probability distribution of the development times for individuals within a population. As insect development duration is temperature-dependent, the cumulative probability distributions are shown over time. The duration of each life stage was indicated as days spent at the designated temperature and standardized by dividing the number of days by the average developmental time at that temperature. To create a distribution model for each life stage independent of temperature, the cumulative proportion was plotted against normalized times. The cumulative proportion was calculated by dividing the cumulative count of individuals completing any life stage at a normalized time by the total number of individuals completing any life stage. This temperature-insensitive distribution can be used to forecast the growth duration of individuals in a population under fluctuating temperature scenarios. Further, this kind of study is also ecologically relevant because it directly influences their geographical distribution, population dynamics, and seasonal phenological timing [52]. The relationship between the cumulative proportion of each life stage and developmental duration [53,54], which aids in forecasting minor data using the Weibull function (Equation (1)), was determined by fitting a two-parameter Weibull distribution function:

(1) $$F\left(t\right)=1-\exp\left[-({\frac{t-\;\gamma }{\alpha })}^{\beta }\right]$$

where *F*(*t*) is the cumulative proportion at normalized time t, and *α*, *β*, and *γ* are parameters of the Weibull function [54].

### 2.5. Developmental Rate Models

The developmental rates of egg, nymph, and egg-to-adult emergence were fitted to linear and nonlinear developmental rate functions. The linear model is expressed as *y* = *aT + b*, where *y* is the rate of development at temperature *T*, *a* is the slope, and *b* is the y-intercept. The lower developmental threshold (LDT) and thermal constant (K) were obtained by calculating the values of −(*b*/*a*) and *1*/*a* of the fitted equation, respectively, for each stage [55].

Among the numerous nonlinear equations proposed to describe the relationship between developmental rate and temperature [56], we selected the Lactin2 model because it best described the asymmetric thermal response, including the decline at supra-optimal temperature, and provided the most biologically interpretable and statistically robust fit to our data [57] (Equation (2)).

(2) $$r\left(T\right)=e^{\rho T}-e^{\left(\rho T_{L}-\frac{\left(T_{L}-T\right)}{∆T}\right)}+\lambda$$

where *r*(*T*) is the developmental rate (1/developmental time) at different constant temperatures (°C); *T* is the absolute temperature (°C); ρ is a constant defining the rate of optimal temperature, $T_{L}$ is the high temperature threshold, ∆T is the temperature range over which physiological breakdown becomes the overriding influence, and λ allows for the estimation of a low-temperature threshold.

### 2.6. Simulation of Adult Emergence

The emergence proportion of *D. baccarum* adults was simulated with respect to constant temperature (°C) and time (day) by combining the nonlinear and Weibull functions (Equation (3)):

(3) $$F\left(x,\;T\right)=1-\exp[-({\frac{xr\left(T\right)-\;\gamma }{\alpha })}^{\beta }]$$

where *F*(*x, T*) is the emergence frequency of *D. baccarum* adults at time *x* and constant temperature *T* (°C), *x* is time (day), *r*(*T*) is the development rate from the selected model, and *α*, *β*, and *γ* are parameters of the Weibull function.

### 2.7. Meteorological Data

Weather data were obtained from the Korean Meteorological Administration (http://data.kma.go.kr/), focusing on the Miryang (study location) meteorological station in Gyeongsangnam Province, Korea. The weather data (annual temperature) were used to develop a degree-day model to estimate the voltinism and spring emergence of *D. baccarum*.

### 2.8. Statistical Analysis

Regression analyses were conducted to model the temperature-dependent development of each *D. baccarum* stage, with model parameter values for both linear and nonlinear functions estimated using the TableCurve 2D v4.0 Automated Curve Fitting program [58]. Data on the life table variables were tested with the Kolmogorov–Smirnov test, assessing whether their normality and homogeneity of variance. Then, the impact of temperature on the developmental duration of each stage, adult lifespan, and % mortality of *D. baccarum* was examined through analysis of variance, ANOVA, using PROC GLM. Tukey’s Studentized Range Honestly Significant Difference (HSD) was utilized for additional differentiation of means among the factors. The variation in adult lifespan between females and males was analyzed using a *t*-test. The ANOVA, *t*-test, and GLM were conducted using SAS software v9.4 [59]. Model adequacy for both linear and nonlinear developmental rate functions was evaluated by examining residual distribution, including checks for normality, homoscedasticity, and temperature-dependent patterns.

## 3. Results

### 3.1. Developmental Period

The developmental periods of eggs, nymphs, and total immature stages of *D. baccarum* were significantly different across temperatures. No first-instar nymphs survived under extreme temperatures (15.3 and 40.0 °C), even though egg development occurred (Table 1 and Tables S2, S3 and S7). The developmental time of *D. baccarum* decreased as temperature increased from 15.3 to 35.1 °C. The mean developmental period required for egg development ranged from 30.56 to 2.07 days at 15.3 to 40.0 °C, respectively. Meanwhile, the development of nymph stages ranged from 64.75 to 21.17 at 20.8 to 35.0 °C, respectively.

### 3.2. Developmental Distribution Model

**Figure 1:**
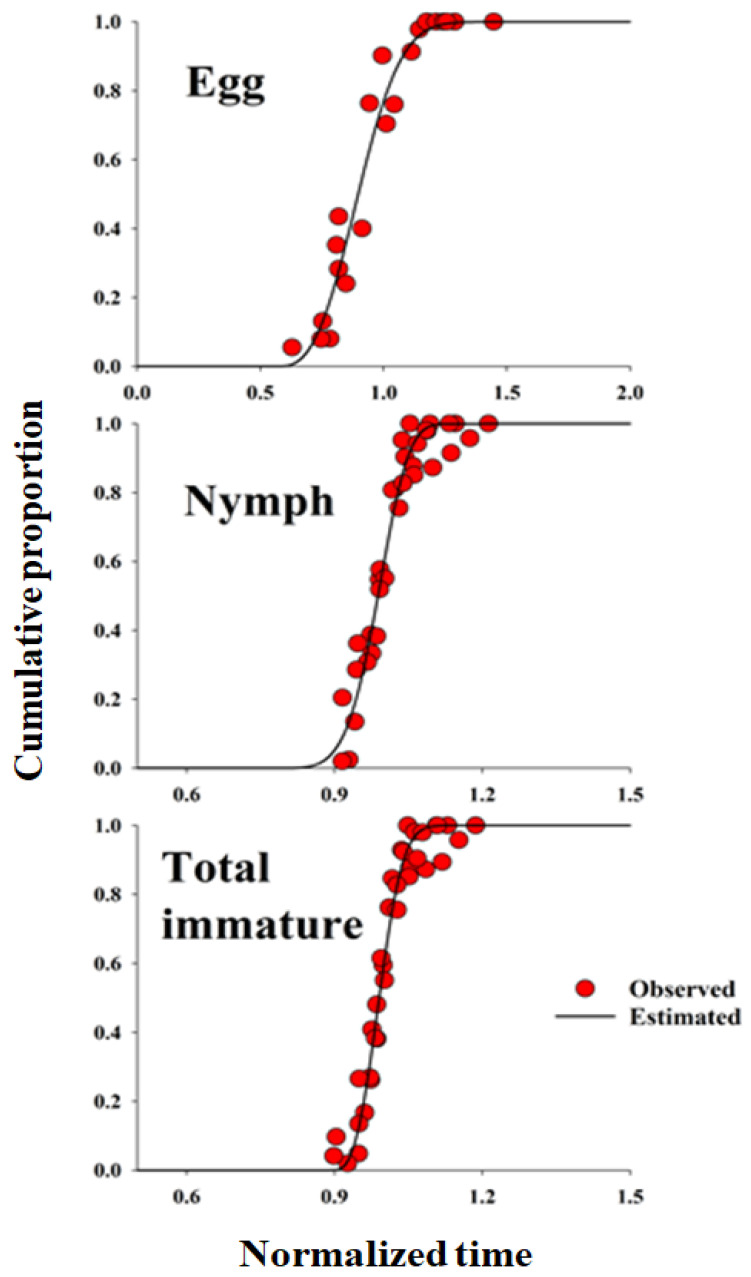
The cumulative proportions of development completion for each life stage of *Dolycoris baccarum* as a function of normalized time (development time/mean developmental time). The observed data were fitted using the three-parameter Weibull function. Parameter estimates for the Weibull distribution used to generate these curves are provided in Table 4.

The frequency distribution of *D. baccarum* developmental stages relative to normalized time (day/median) on sesame seed pods was described using the Weibull model (Figure 1). The cumulative frequency distribution of *D. baccarum* development time at different temperatures on the sesame seed pod was accurately represented by the Weibull model (R^2^ > 0.94, *p* < 0.0001). A linear regression model was used to describe the relationship between temperature and the developmental rates of various life stages of *D. baccarum,* and was applied to the mid-range development rates of sesame seed pods. The LDT and K values for each developmental stage of *D. baccarum* (egg, nymph, and overall immature stage development) were calculated using a regression model (Table 2).

**Figure 2:**
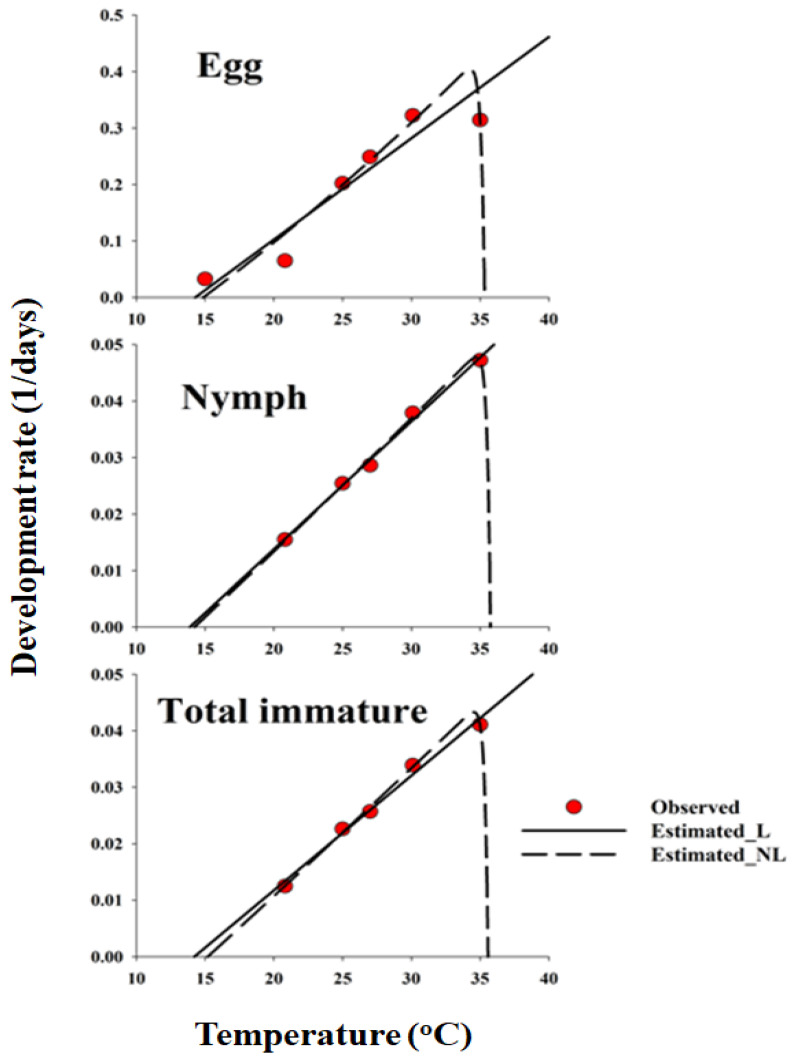
Linear and nonlinear functions fitted to the data of developmental rate (day-1) for each life stage of *Dolycoris baccarum*. In the egg stage, the observation data for 40 °C were excluded from the nonlinear function model. L and NL denote linear and nonlinear models, respectively. Total immature refers to the combined developmental period from egg to adult emergence. Parameter estimates for the nonlinear Lactin2 curves shown in the figure are provided in Table 3.

The parameters estimated for the nonlinear function for each life stage of sesame seed pods are listed in Table 3. A typical skewed bell-shaped curve was observed, showing a steep decline at temperatures exceeding the optimum, illustrating the temperature-dependent pattern of *D. baccarum* development rates across the full temperature range (Figure 2). The nonlinear model (Lactin2) was identified as the most suitable representation of the temperature-dependent rate of *D. baccarum* development in sesame seed pods (Figure 2 and Table 3). However, the linear GLM provided a slightly better fit for the nymphal developmental rates within the mid-temperature ranges, whereas the nonlinear Lactin-2 model more accurately described the full thermal response for eggs and total immature development. The temperature range for the development of *D. baccarum* was estimated from *T_L_* to Δ*T*. Residual plots were inspected to verify that model assumptions were met and that no systematic pattern remained in the residuals.

**Table 3:** Estimated parameter values of the Lactin2 model against the development rate for each stage of *Dolycoris baccarum*.

Parameters	Life Stage		
	Egg	Nymph	Total Immature
*ρ*	0.0147	0.0022	0.0021
*T_L_*	35.6654	36.4538	36.3348
Δ*T*	0.2466	0.2266	0.2364
*λ*	−1.2431	−1.0323	−1.0331
*r* ^2^	0.96	0.99	0.99

Egg: F_3, 2_ = 14.42, *p* < 0.0653; Nymph: F_3, 2_ = 187.18, *p* < 0.0053; Total immature: F_3, 2_ = 265.30, *p* < 0.0038.

**Table 4:** Parameter estimates of nonlinear developmental rate models of *Dolycoris baccarum* at different temperatures.

Parameters	Life Stage		
	Egg	Nymph	Total Immature
*α*	0.3681	0.2335	0.105
*β*	2.6911	4.9538	2.7852
*γ*	0.5758	0.7716	0.8988
*r* ^2^	0.95	0.96	0.97

Egg: F_2, 19_ = 183.86, *p* < 0.0001; Nymph: F_2, 30_ = 320.54, *p* < 0.0001; Total immature: F_2, 32_ = 467.75, *p* < 0.0001.

### 3.3. Simulation of Adult Emergence

**Figure 3:**
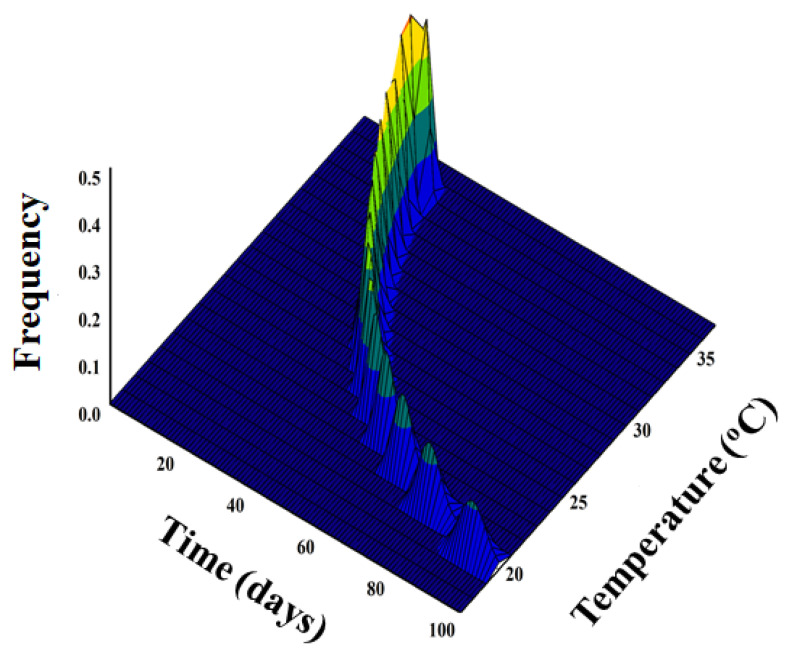
Simulated temperature-dependent adult emergence of *Dolycoris baccarum* from egg cohort using nonlinear function in relation to time (day) and temperature (°C).

The nonlinear function was applied to predict the daily emergence of adult *D. baccarum* from a cohort of eggs under different temperatures and is illustrated with respect to temperature (°C) and time (days) (Figure 3). Adult emergence occurred more quickly over a much briefer timeframe at optimal temperatures. However, adult emergence was postponed when exposed to prolonged durations of extremely low temperatures. For instance, adult emergence on the sesame seed pods occurred between 20 and 30 days at 35.1 #xB0;C, 25 and 30 days at 27.0 °C, and 90 and 97 days at 20.8 °C.

### 3.4. Voltinism and Spring Emergence

Based on the biofix date (1 January), the voltinism of *D. baccarum* on sesame seed pods varied between 2.58 and 3.16 generations over a seven-year period. The count of generations was nearly the same, with a marginal increase (3.77) in 2024. In the field, adult emergence on sesame seed pods during spring occurred between 27 June and 5 July over the seven-year period. Early spring emergence occurred on 27 June 2020, whereas late spring emergence occurred on 5 July 2019 (Table 5 and Table S1).

### 3.5. Adult Longevity and Sex Ratio

The lifespan of adult *D. baccarum* varied significantly with temperature, and the lifespans of females and males also differed across temperature treatments (Table 6 and Tables S4 and S5). Adults lived the longest at 30.1 °C (female: 44.16 and male: 36.10 days), followed by 27.0 °C (female: 37.80 and male: 34.74 days). Notably, shorter lifespans were observed at 20.8 °C for females (28.81 days) and at 35.0 °C for males (21.35 days). Female bugs that emerged from the sesame seed pod lived longer than male bugs under all temperature regimes. The female proportion of *D. baccarum* did not differ among temperatures (*p* > 0.05). The female/male ratio was approximately 1:1 at the tested temperatures. The highest female ratio (0.69) was recorded at 25.0 °C, and the lowest (0.43) was recorded at 20.8 °C.

### 3.6. Mortality

The mortality of each developmental stage of *D. baccarum* varied significantly across temperatures (Table 7 and Table S6). Egg mortality decreased with increasing temperature, with no mortality recorded at 27.0 and 30.1 °C. The highest nymph mortality (100 and 100%) occurred under extreme temperatures of 15.3 and 40.0 °C, respectively. The lowest mortality at immature stages (eggs and nymphs) was observed at mid-range temperatures (25.0 to 30.1 °C). Minimum adult mortality was observed between temperatures ranging from 25.0 to 35.0 °C, with a slightly higher rate (8.62%) at 20.8 °C.

## 4. Discussion

We estimated the thermal requirements for the development of *D. baccarum* at different temperatures to assess the relationship between temperature and the developmental processes of *D. baccarum*. The findings demonstrate that temperature is one of the most important environmental factors determining the developmental processes of insects. The developmental phenomenon of our study shows that the developmental time of the *D. baccarum* population decreased significantly as temperature increased (Table 1). Moreover, this supports the idea that temperature significantly influences developmental time and accelerates the development of several insect species, including *D. baccarum,* under rising temperature conditions. This aligns with previous studies that have identified temperature as one of the most influential abiotic factors affecting the overall developmental processes of insects [21,22,60,61]. Therefore, these results contribute to the development of forecasting models, the estimation of spring adult emergence in the field, and the formulation of integrated management strategies for hemipterans, including *D. baccarum*. Moreover, this study provides insight into how global warming-mediated temperature increases influence the biological processes and population dynamics of flora and fauna. These climatic-driven changes are considered among the most significant challenges to pest management and food security worldwide [16,62,63].

In this study, we estimated the thermal requirements of *D. baccarum* using linear and nonlinear developmental rate models on sesame seed pods and found that the development of eggs and nymphs was shorter at higher temperatures and longer at lower temperatures. This developmental pattern is largely in agreement with previous studies [13,41,64]. We estimated total development times of 80.09 at 20.8 °C and 44.26 days at 25.0 °C, which are slightly higher than the estimates (65 days) at 20 °C reported by [41] and (28.9 days) at 25.0 °C by [13]. This inconsistency may arise from differences in the genetic makeup of the *D. baccarum* population, experimental setup or methodology, and food sources. In this study, the developmental period of *D. baccarum* on sesame seed pods was examined, in contrast to previous studies [41] and [13] which used raw peanuts as food sources. Therefore, this observed variation may have affected the available nutrient content of the food sources provided [30,31]. Refs. [30,31] performed nutrient content analysis of different host plants of Spodoptera species and found that nutrient content significantly varied among host plants, which further affected the developmental time of *S. exigua* and *S. litura*. At extreme temperatures (15.3 and 40.0 °C), none of the nymph stages survived. This unsuccessful development and the subsequent mortality of nymphs may be attributed to rapid dehydration and physiological stress induced by exposure to cold and hot temperatures. This explanation is supported by Van der Have [65], MacQuarrie et al. [66], and Neven [67], who reported that prolonged exposure to extreme temperatures causes metabolic disorders and insect death by disrupting biological processes. Certain temperature-related events produce stressful environmental conditions that disrupt normal insect development, potentially causing mortality (especially in immature stages) due to significant tissue damage from cold stress or accumulated damage that leads to metabolic disruptions, even at temperatures above freezing [68,69]. Insect development and mortality also depend on the freeze tolerance and supercooling point of each insect species. Ref. [70] mentioned that freeze-tolerant insects raise their supercooling point for winter to trigger freezing at elevated temperatures, while freeze-avoiding insects endure winter in a supercooled condition by lowering their supercooling point. Meanwhile, when temperatures rise above a critical level, the enzymes essential for metabolic and physiological functions become denatured, halting developmental processes [71]. In addition to the impact on metabolic and physiological functions, the exposure duration to temperature extremes may also influence developmental variables. Ref. [72] reported that prolonged exposure to thermal extremes during various developmental stages generally affects insect life history, reducing egg hatching and larval survival to adulthood, and may also cause deformities in adult insects. Refs. [73,74] reported that thermal tolerance and physiological adjustments of insects also impact the development and survival of insect species. However, this type of mortality varied depending on the rearing conditions (laboratory-reared vs. outdoor field-collected insects) [75]. Therefore, nymphal development failure at constant extreme temperatures in the laboratory may not occur in the field due to fluctuating temperatures. In addition to affecting the life variables of insects, temperature also affects host plant physiology, indirectly altering the development of insect species [76]. Apart from temperature, the photoperiod also influences the development, reproduction, oviposition, and diapause of *D. baccarum* [10,13,41]. Ref. [41] reported that developmental time was significantly extended at temperatures of 20 or 25 °C with an extended light duration; however, when the light duration was extended to 16L:8D and 18L:6D, the developmental time was significantly reduced. In addition, the developmental time was notably reduced with higher temperatures, with a photoperiod of 12L:12D for short days and 16L:8D for long days. In addition, Ref. [13] reported the impact of the photoperiod and temperature on the growth rate, developmental time, and adult size of *D. baccarum*.

In the present study, we found that temperature plays a critical role in regulating the lifespan of *D. baccarum*. However, the proportion of female *D. baccarum* did not vary, and almost equal numbers of each sex emerged when nymphs were fed with sesame seed pods under the tested temperature regimes, with a slight decrease at 20.8 °C (Table 6). Longevity variations seen among temperatures may also be attributed to the nutritional quality and quantity of the host plant, as well as the temperature. This was presented in Refs. [30,31], which reports the relationships between life variables and host plants and their effects on the development and longevity of *Spodoptera* sp. Having said that, we suspect that temperature has played an important role in the lifespan of *D. baccarum* in this study as the insects received the same food source at all tested temperatures. In addition to the nutritional quality of host plants, differences in longevity may be associated with various factors and interactions, such as the intricate relationship between particular native environmental conditions and sex-specific reproductive costs, epigenetic regulation of longevity through DNA methylation imprinting, and enhanced fecundity and aging protection resulting from mating or elements of the male ejaculate [77].

A population growth assessment of organisms is typically performed using origin and development-based minimum temperature thresholds [78]. Kiritani [79] proposed that utilizing the LDT (*T*o) and thermal constant (K) reported for insects can help forecast the phenology of insect communities in the context of global warming. In this study, we assessed the effects of LDT and K on the development of *D. baccarum* using a linear model derived from sesame seed pods. The analysis of the linear model was used to estimate the LDT (14.22 °C) and K (492.22 DD) values so we could understand the development of *D. baccarum* under varied temperatures (Table 2). The LDT we calculated was nearly identical to the LDT (13.2 °C) reported by [80,81] for Pentatomidae species. However, the estimated K value (492.22 DD) in this study differed from the estimation of Kiritani (447.11 DD for the Pentatomidae species) [81,82]. The LDT that we estimated is in agreement with the LDT estimated by Ahn [82] for Hemipterans, who reported that 14.1 °C resulted in total immature stage development. The variations in the thermal requirements for development could be attributed to several factors, such as the conditions under which the bugs are reared, the environmental factors they are exposed to, available food types (plant/seed/host/artificial diet), nutrient compositions of host plants, and the overall configurations of the experiments. Therefore, it is important to consider whether the materials and methods employed affect the developmental needs of *D. baccarum*.

The Intergovernmental Panel of Climate Change [83] states that the majority of the warming estimated over the past 50 years is a result of human actions. The worldwide average surface temperature is estimated to rise by 1.4–5.8 °C between 1990 and 2100. The IPCC indicated that a global temperature increase of roughly 2 °C in the coming century will cause adverse impacts, affecting the majority of areas worldwide. The most predictable effects of climate change will significantly influence the behavior and population dynamics of herbivorous insects; typically, the annual number of generations is a crucial factor influencing multivoltine species populations [84]. Moreover, temperature-driven extended seasons for reproduction and reduced diapause could lead to more generations each year or enhanced success in overwintering [70,73,74]. In this study, we observed approximately three generations annually in Korea, based on the estimated thermal data of *D. baccarum* (Table 5). This is consistent with the findings of Nakamura and Numata [10], who reported that *D. baccarum* can produce three generations per year in Japan. Considering the climate change trends observed in recent years, Kiritani [85] noted the likelihood of an increase in the number of generations (1.02) of Pentatomidae insects. The potential for an increase in generations in Korea cannot be overlooked, as temperature fluctuations resulting from climate change are already evident, with the average temperature rising by approximately 1.0 °C over the past three decades [34]. Moreover, the impacts of climate change have already been observed in Miryang as winter temperatures increase [86], and earlier insect emergence in spring has been recorded [87].

In the present study, we calculated the minimum temperature threshold and thermal constant of *D. baccarum* using linear models, which are widely employed to evaluate the lower temperature limit and thermal constant of insect species [88], despite several limitations pointed out by analysts [89,90]. These models remain popular due to their simplicity, requiring minimal data inputs for definition through straightforward calculations. Moreover, their general use has been determined to be adequate, with satisfactory precision [91]. Utilizing a linear model with thermal constant estimates for sesame seed pods, we evaluated the straightforward application of a degree-day model with a biofix on 1 January [92,93] to forecast the spring emergence dates for *D. baccarum* in sesame fields in Korea were 1 July 2018, 5 July 2019, 27 June 2020, 4 July 2021, 28 June 2022, 1 July 2023, and 29 June 2024 (Table 5) (Maharjan, unpublished). As a result, this study may aid in understanding the temperature-sensitive growth of this polyphagous pest in Korea and is expected to be useful for predicting shifts in pest status, range of distribution expansion, winter mortality rates, generation numbers, and population control of *D. baccarum* with respect to climate change effects.

## 5. Conclusions

Our estimates of the developmental thermal requirements, developmental time, mortality, and adult lifespan of *D. baccarum* in the Korean local population enhance the current life history data regarding this species, and are applicable to integrated pest management (IPM). In particular, our estimates lay the groundwork for creating degree-day models and more intricate mechanistic phenology and population models for the local Korean population which can be utilized to predict the growth rates, spring emergence, and population dynamics of *D. baccarum* in Korea. Population growth may be examined through different climate change scenarios pertinent to the southern areas of the country or in response to IPM strategies. Future studies should also focus on validating temperature-dependent models related to the development, reproduction, and survival of *D. baccarum* populations in natural environments as they respond to variable temperatures. The phenological model created for this pest should be enhanced with updated data regarding temperature-dependent fecundity and lifespan, and integrated with a phenological model designed for primary crops that are significantly at risk from this pest. By linking crop models developed from our thermal estimates with pest models, stakeholders, such as IPM extension personnel, farm managers, and crop advisors, will be able to create and execute cost-effective, environmentally sustainable pest control strategies for this species. This approach will help minimize ecological harm by providing a better understanding of how pest dynamics respond to changing climatic conditions.

## Figures and Tables

**Table 1:** Developmental period (days, mean ± SE) of immature stages (egg–adult) of *Dolycoris baccarum* on sesame seed pod as food source at different temperatures.

Life Stage	Nymph Stage	Temperature (°C)						
		15.3	20.8	25.0	27.0	30.1	35.0	40.0
Egg	-	30.56 ± 0.8 a	15.34 ± 0.3 b	4.94 ± 0.1 c	4.02 ± 0.1 c	3.10 ± 0.0 d	3.18 ± 0.1 d	2.07 ± 0.0 e
Nymph	1st	Dead	9.58 ± 0.2 a	5.16 ± 0.1 b	3.69 ± 0.1 c	3.20 ± 0.1 d	2.88 ± 0.1 e	Dead
	2nd	-	2.30 ± 0.1 d	7.20 ± 0.3 a	6.49 ± 0.2 b	3.48 ± 0.1 c	3.39 ± 0.1 c	-
	3rd	-	18.27 ± 0.6 a	8.41 ± 0.3 b	6.81 ± 0.3 c	5.04 ± 0.2 d	4.16 ± 0.1 e	-
	4th	-	5.14 ± 0.2 d	7.84 ± 0.3 b	9.30 ± 0.4 a	6.13 ± 0.4 c	4.25 ± 0.1 e	-
	5th	-	29.46 ± 0.2 a	10.71 ± 0.7 b	8.64 ± 0.4 c	8.54 ± 0.1 c	6.49 ± 0.3 d	-
Total (1–5 stage)	-	-	64.75 ± 0.2 a	39.32 ± 0.3 b	34.93 ± 0.3 c	26.39 ± 0.4 d	21.17 ± 0.2 e	-
Total (egg-adult)	-	-	80.09 ± 0.3 a	44.26 ± 0.2 b	38.95 ± 0.3 c	29.49 ± 0.4 d	24.35 ± 0.2 e	-

Initial egg no.: 49 at 15.3 °C, 58 at 20.8 °C, 55 at 25 °C, 51 at 27 °C, 49 at 30.1 °C, 56 at 35 °C, and 74 at 40 °C. ANOVA for Egg: F_6, 360_ = 1528.59, *p* < 0.0001; First-instar nymphs: F_4, 229_ = 688.53, *p* < 0.0001; Second-instar nymphs: F_4, 227_ = 94.86, *p* < 0.0001; Third-instar nymphs: F_4, 227_ = 331.16, *p* < 0.0001; Fourth nymph stage: F_4, 227_ = 47.25, *p* < 0.0001; Fifth nymph stage: F_4, 227_ = 374.76, *p* < 0.0001; Total nymph (1–5 instar): F_4, 237_ = 2893.18, *p* < 0.0001; Total (egg-adult): F_4, 237_ = 4552.16, *p* = 0.0364. Means followed by the same letters in a row are not significantly different between temperatures (ANOVA, Tukey’s HSD test, *p* < 0.05). SE: Standard error.

**Table 2:** Linear regression analysis of pre-adult development, lower developmental threshold (LDT), and thermal constant (K) for *Dolycoris baccarum*.

Life Stage	Linear Regression	*r* ^2^	LDT (°C)	K (DD)
Egg	0.0179 T–0.2559	0.94	14.27	55.76
Nymph	0.0023 T–0.0315	0.99	13.90	442.08
Total immature	0.0020 T–0.0289	0.99	14.22	492.22

T indicates test temperature. Egg: F_1, 5_ = 77.78, *p* < 0.0003; Nymph: F_1, 3_ = 596.02, *p* < 0.0001; Total immature: F_1, 3_ = 263.06, *p* < 0.0005.

**Table 5:** Estimated annual voltinism over a seven-year period in Miryang, Korea, based on a biofix date of 1 January and spring emergence dates of *Dolycoris baccarum* adults.

Year	Food Source	Biofix of 1 January	
		Thermal Constant (DD)	Emergence Date
2018	Sesame	496.59	1 July
2019	Sesame	501.93	5 July
2020	Sesame	496.96	27 June
2021	Sesame	500.12	4 July
2022	Sesame	496.74	28 June
2023	Sesame	501.69	1 July
2024	Sesame	499.08	29 June

Spring emergence dates for *Dolycoris baccarum* adults were predicted from the degree-day calculation using the same weather data for each year for host plants (sesame) based on the lower developmental threshold (LDT).

**Table 6:** Adult longevity (days, mean ± SE) of immature stages (egg–adult) and sex ratio (female proportion) of *Dolycoris baccarum* on sesame seed pod as food source at different temperatures.

Parameters	Sex	Temperature (°C)						
		15.3	20.8	25.0	27.0	30.1	35.0	40.0
Longevity	Female	-	28.81 ± 0.8 e, *	35.15 ± 0.3 c, *	37.80 ± 0.3 b, *	44.16 ± 0.6 a, *	31.52 ± 1.3 d, *	-
	Male	-	24.38 ± 0.4 c	32.28 ± 0.4 b	34.74 ± 0.3 a	36.10 ± 1.3 a	21.35 ± 0.9 d	-
Sex ratio	-	-	0.43	0.69	0.52	0.54	0.47	-

ANOVA for female: F_4, 110_ = 66.76, *p* < 0.0001 and male: F_4, 111_ = 84.94, *p* < 0.0001. T-test for 20.8 °C: t_15_ = 5.93, *p* < 0.0001; 25 °C: t_24_ = 5.28, *p* < 0.0001; 27 °C: t_22_ = 6.70, *p* < 0.0001; 30.1 °C: t_20_ = 6.53, *p* < 0.0001; 35 °C: t_22_ = 10.28, *p* < 0.0001. Means followed by the same letters in a row are not significantly different among temperatures (ANOVA, Tukey’s HSD test, *p* < 0.05). * Significantly different between sexes (*t*-test, *p* < 0.05). SE: Standard error.

**Table 7:** Mortality (%, mean ± SE) of different stages of *Dolycoris baccarum* on sesame seed pod as food source at different temperatures.

Life Stage	Temperature (°C)						
	15.3	20.8	25.0	27.0	30.1	35.0	40.0
Egg	20.41 ± 5.9 a	13.79 ± 4.6 b	1.82 ± 1.4 d	0.00 ± 0.0	0.00 ± 0.0	1.79 ± 1.5 d	6.76 ± 2.7 c
Nymph	100 ± 0.0 a	13.79 ± 4.5 b	3.64 ± 2.6 d	5.88 ± 3.4 c	4.08 ± 2.9 d	5.36 ± 3.1 c	100 ± 0.0 a
Adult	-	8.62 ± 3.8 a	1.82 ± 1.5 b	1.96 ± 0.9 b	2.04 ± 1.9 b	1.79 ± 1.5 b	-

ANOVA for Egg: F_6, 385_ = 5.75, *p* < 0.0001; Nymph: F_6, 385_ = 124.84, *p* < 0.0001; Adult: F_6, 385_ = 2.27, *p* = 0.0364. Means followed by the same letters in a row are not significantly different between temperatures (ANOVA, Tukey’s HSD test, *p* < 0.05). SE: Standard error. - Hyphen inside table indicates no adult development due to nymph mortality.

## Data Availability

The original contributions presented in this study are included in the article. Further inquiries can be directed to the corresponding authors.

## Supplementary Materials

The following supporting information can be downloaded at: https://www.mdpi.com/article/10.3390/insects16121245/s1.

## References

[1] Chung B.K., Kang S.W., Kwon J.H. (1995). Damage, occurrence, and control of hemipterous insects in non-astringent persimmon orchards. RDA J. Agric. Sci..

[2] Leal W.S., Higuchi H., Mizutani N., Nakamori H., Kadosawa T., Ono M. (1995). Multifunctional communication in *Riptortus clavatus* (Heteroptera: Alydidae) conspecific nymph and egg parasitoid *Ooencyrtus nezarae* use the same adult attractant pheromone as chemical cue. J. Chem. Ecol..

[3] Jansen R.L., Newsom L.D. (1972). Effect of stink bug damaged soybean seeds on germination, emergence, and yield. J. Econ. Entomol..

[4] Son C.K., Park S.G., Hwang Y.H., Choi B.S. (2000). Field occurrence of stink bug and its damage in soybean. Korean J. Crop Sci..

[5] Choi M.Y., Lee G.H., Park C.H., Seo H.Y., Oh Y.J., Kim D.H., Kim J.D. (2005). Feeding preference, nymphal development time, body weight increase, and survival rate of the bean bug, *Riptortus clavatus* (Thunberg) (Hemiptera: Alydidae) on soybean varieties. Korean J. Appl. Entomol..

[6] Daughtery D.M. (1967). Pentatomidae as vectors of yeast spot diseases of soya beans. J. Econ. Entomol..

[7] Bae S.D., Kim H.J., Park S.T. (2012). Attractiveness of conspecific stink bugs to adult stink bug-baited traps in soybean fields. J. Asia-Pac. Entomol..

[8] Panizzi A.R., McPherson J.E., James D.G., Javahery M., McPherson R.M., Schaefer C.W., Panizzi A.R. (2000). Stink bugs (Pentatomidae). Heteroptera of Economic Importance.

[9] Kobayashi T. (1972). Biology of insect pests of soybean and their control. Jarq.

[10] Nakamura K., Numata H. (2006). Effect photoperiod and temperature on the induction of adult diapause in *Dolycoris baccarum* (L.) (Heteroptera: Pentatomidae) from Osaka and Hokkaido, Japan. Appl. Entomol..

[11] Tsagkarakis A., Thanou Z., Chaldeou A., Moschou I., Kalaitzaki A., Drosopoulos S. (2022). New records and updated checklist of the Pentatomoidea (Hemiptera: Heteroptera) of Greece. Insects.

[12] Robert N.M.D., Emily C.O., James F.W., Anne L.N. (2021). Diapause termination in invasive populations of the brown marmorated stink bug (Hemiptera: Pentatomidae) in response to photoperiod. Environ. Entomol..

[13] Nakamura K. (2002). Effect of photoperiod on the size-temperature relationship in a Pentatomid bug, *Dolycoris baccarum*. J. Therm. Biol..

[14] Rahmstorf S., Cazenave A., Church J.A., Hansen J.E., Keeling R.F., Parker D.E., Somerville R.C.J. (2007). Recent climate observations compared to projections. Science.

[15] Solomon S., Qin D., Manning M., Chen Z., Marquis M., Averyt K.B., Tignor M., Miller H.L., IPCC (2007). Summary for policymakers. Climate Change 2007: The Physical Science Basis. Contribution of Working Group I to the Fourth Assessment Report of the Intergovernmental Panel on Climate Change.

[16] Walther G.-R., Post E., Convey P., Menzel A., Parmesan C., Beebee T.J.C., Fromentin J.-M., Hoegh-Guldberg O., Bairlein F. (2002). Ecological responses to recent climate change. Nature.

[17] Post E., Stenseth N.C., Langvatn R., Fromentin J.-M. (1997). Global climate change and phenotypic variation among red deer cohorts. Proc. Biol. Sci..

[18] Parmesan C., Yohe G. (2003). A globally coherent fingerprint of climate change impacts across natural systems. Nature.

[19] Hofstetter R.W., Dempsey T.D., Klepzig K.D., Ayres M.P. (2007). Temperature-dependent effects on mutualistic, antagonistic, and commensalistic interactions among insects, fungi and mites. Community Ecol..

[20] Sagarin R.D., Barry J.P., Gilman S.E., Baxter C.H. (1999). Climate-related change in an intertidal community over short and long time scales. Ecol. Monogr..

[21] Taylor F. (1981). Ecology and evolution of physiological time in insects. Am. Nat..

[22] Pedigo L.P. (1989). Entomology and Pest Management.

[23] Briere J.F., Pracros P., Le Roux A.Y., Pierre J.S. (1999). A novel rate model of temperature-dependent development for arthropods. Environ. Entomol..

[24] Angilletta M.J. (2009). Thermal Adaptation: A Theoretical and Empirical Synthesis.

[25] Yang P.J., Carey J.R., Dowell R.V. (1994). Temperature influence on the development and demography of *Bactrocera dorsalis* (Diptera: Tephritidae) in China. Environ. Entomol..

[26] Dreyer H., Baumagartner J. (1996). Temperature influence on cohort parameters and demographic characteristics of the two cow-pea coreids *Clavigralla tomentosicollis* and *C. shadabi*. Entomol. Exp. Appl..

[27] Infante F. (2000). Development and population growth rates of *Prorops nasuta* (Hym., Bethylidae) at constant temperatures. J. Appl. Entomol..

[28] Atkinson D. (1994). Temperature and organism size a biological law for ectotherms?. Adv. Ecol. Res..

[29] Atkinson D., Johnson I.A., Bennett A.F. (1996). Ectotherm life-history responses to developmental temperatures. Animals and Temperature.

[30] Maharjan R., Ahn J., Yi H. (2022). Interactive effects of temperature and plant host on the development parameters of *Spodoptera exigua* (Hübner) (Lepidoptera: Noctuidae). Insects.

[31] Maharjan R., Hong S., Ahn J., Yoon Y., Jang Y., Kim J., Lee M., Park K., Yi H. (2023). Temperature and host plant impacts on the development of *Spodoptera litura* (Fabricius) (Lepidoptera: Noctuidae): Linear and Nonlinear modeling. Insects.

[32] Jarvis C.H., Baker R.H.A. (2001). Risk assessment for nonindigenous pests: 2. Accounting for inter-year climate variability. Diver. Distrib..

[33] Stocker T.F., Qin D., Plattner G.K., Tignor M., Allen S.K., Boschung J., Nauels A., Xia Y., Bex V., Midgley P.M., IPCC (2013). Climate Change: The physical science basis. Contribution of Working Group I to the Fifth Assessment Report of the Inter-Governmental Panel on Climate Change.

[34] Kwon M., Kim J., Maharjan R., Choi J.-Y., Kim G.-H. (2017). Change in the distribution of the potato tuber moth, *Phthorimaea operculella* (Zeller) (Lepidoptera: Gelechiidae) in Korea. J. Asia-Pac. Entomol..

[35] Gregg P.C. (1982). A simulation model of the development of *Chortoicetes terminifera* (Orthoptera: Acrididae) under fluctuating temperatures. Proceedings of the 3rd Australasian Conference Grassland Invertebrate Ecology.

[36] Hodek I. (1977). Photoperiodic response in spring in three Pentatomoidea (Heteroptera). Acta Entomol. Bohemoslov..

[37] Babrakzai Z.H., Hodek I. (1987). Diapause induction and termination in a population of *Dolycoris baccarum* (Heteroptera: Pentatomoidea) from Central Bohemia. Věstník Ceskoslov. Společnosti Zool..

[38] Karsavuran Y. (1986). Investigations on the biology and ecology of *Dolycoris baccarum* (L.) (Het.: Pentatomidae) which attacks to the various plants of economic importance at Bornova (Izmir.). Turk. Bitki Kor. Derg..

[39] Hodkova M., Hodek I., Somme L. (1989). Cold is not a prerequisite for the completion of photoperiodically induced diapause in *Dolycoris baccarum* from Norway. Entomol. Exp. Appl..

[40] Hodek I., Hodkova M. (1993). Role of temperature and photoperiod in diapause regulation in Czech populations of *Dolycoris baccarum* (Heteroptera: Pentatomoidea). Eur. J. Entomol..

[41] Tian R., Hou Z., Li S., Chai H. (2024). Effects of photoperiod and temperature on the developmental duration and diapause in *Dolycoris baccarum* (Heteroptera: Pentatomidae) from Hohhot, Inner Mongolia, China. J. Insect Sci..

[42] Nielsen A.L., Hamilton G.C., Matadha D. (2008). Developmental rate estimation and life table analysis for *Halyomorpha halys* (Hemiptera: Pentatomidae). Environ. Entomol..

[43] Zerbino M.S., Altier N.A., Panizzi A.R. (2013). Effect of photoperiod and temperature on nymphal development and adult reproduction of *Piezodorus guildinii* (Heteroptera: Pentatomidae). Fla. Entomol..

[44] Da Silva P.G., Daane K.M. (2014). Life history parameters of *Chinavia hilaris* (Hemiptera: Pentatomidae), a stink bug injurious to pistachios in California. J. Econ. Entomol..

[45] Jung K.Y., Lak A.S. (1985). Studies on the insect pests of barley in Korea. Curr. Res. Agric. Life Sci..

[46] Bae S.D., Kim H.J., Lee G.H., Park S.T., Lee S.W. (2008). Susceptibility of stink bugs collected in soybean fields in Milyang to some insecticides. Korean J. Appl. Entomol..

[47] Kim K.Y., Choi D.S., Choi J.-Y., Hong K.-J. (2017). Host records of *Trissolcus* (Hymenoptera: Platygasteridae: Telenominae) parasitizing eggs of stink bugs in Korea. Korean J. Appl. Entomol..

[48] Mahmoud A.M.A., Lim U.T. (2007). Evaluation of cold-stored eggs of *Dolycoris baccarum* (Hemiptera: Pentatomidae) for parasitization by *Trissolcus nigripedius* (Hymenoptera: Scelionidae). Biol. Control..

[49] Shim K.B., Hwang C.-D., Pae S.-B., Lee C.-K., Kim S.-U., Park C.-H., Lee M.-H., Jeong C.S., Park K.-Y., Song D.-Y. (2013). A new white sesame variety, ‘Milsung,’ has a dense capsule and a high yield. Korean J. Breed. Sci..

[50] Barghi N., Ramirez-Lanzas C. (2023). A high throughput method for egg size measurement in Drosophila. Sci. Rep..

[51] Winston P.W., Bates D.H. (1960). Saturated solutions for the control of humidity in biological research. Ecology.

[52] Régnière J., Powell J., Bentz B., Nealis V. (2012). Effects of temperature on development, survival and reproduction of insects: Experimental design, data analysis and modeling. J. Insect Physiol..

[53] Wagner T.L., Wu H.I., Sharpe P.J.H., Schoolfield R.M., Coulson B.N. (1984). Modeling insect development rates: A literature review and application of a biophysical model. Ann. Entomol. Soc. Am..

[54] Weibull W. (1951). A statistical distribution functions with wide applicability. J. Appl. Mech..

[55] Campbell A., Frazer B.D., Gilbert N., Gutierrez A.P., Mackauer M. (1974). Temperature requirements of some aphids and their parasites. J. Appl. Ecol..

[56] Kim D.-S., Ahn J.J., Lee J.-H. (2017). A review for non-linear models describing temperature-dependent development of insect populations: Characteristics and developmental process of models. Korean J. Appl. Entomol..

[57] Lactin D.J., Holliday N.J., Johnson D.L., Craigen R. (1995). Improved rate model of temperature-dependent development by arthropods. Environ. Entomol..

[58] Jandel Scientific (1994). TableCurve User’s Manual.

[59] SAS, Institute Inc (2002). SAS Use’s Guide. Statistics.

[60] Lang H., Okland B., Krokene P. (2006). Thresholds in lifecycle of the spruce bark beetle under climate change. Interj. Complex Syst..

[61] Maharjan R., Hong S., Yoon Y., Jang Y., Park K. (2024). Temperature and relative humidity mediated life processes of *Spodoptera* sp. (Lepidoptera: Noctuidae). Bull. Entomol. Res..

[62] Penuelas J., Filella I. (2001). Phenology. Responses to a warming world. Science.

[63] Root T.L., Price J.T., Hall K.R., Schneider S.H., Rosenzweig C., Pounds J.A. (2003). Fingerprints of global warming on wild animals and plants. Nature.

[64] Mohammed A.M.A., Abdulla B.S., Abdurrahman N.M. (2017). The biological study on *Dolycoris baccarum* (Linnaeus) (Heteroptera: Pentatomidae) in Erbil-Kurdistan Region-Iraq. Iraqi J. Agric. Sci..

[65] Van der Have T.M. (2002). A proximate model for thermal tolerance in ectotherms. Oikos.

[66] MacQuarrie C.J.K., Derry V., Gray M., Mielewczyk N., Crossland D., Ogden J.B., Boulanger Y., Fidgen J.G. (2024). Effect of a severe cold spell on overwintering survival of an invasive forest insect pest. Curr. Res. Insect Sci..

[67] Neven L.G. (2000). Physiological responses of insects to heat. Postharvest Biol. Technol..

[68] Turnock W.J., Fields P.G. (2005). Winter climates and cold hardiness in terrestrial insects. Eur. J. Entomol..

[69] Williams C.M., Henry H.A.L., Sinclair B.J. (2015). Cold truths: How winter drives responses of terrestrial organisms to climate change. Biol. Rev..

[70] Rozsypal J., Košťál V. (2018). Supercooling and freezing as eco-physiological alternatives rather than mutually exclusive strategies: A case study in *Pyrrhocoris apterus*. J. Insect Physiol..

[71] Daniel R.M., Danson M.J. (2013). Temperature and the catalytic activity of enzymes: A fresh understanding. FEBS Lett..

[72] Martins J.C., Picanço M.C., Bacci L., Guedes R.N.C., Santana P.A., Ferreira D.O., Chediak M. (2016). Life table determination of thermal requirements of the tomato borer *Tuta absoluta*. J. Pest Sci..

[73] Košťál V., Šimek P. (2000). Overwintering strategy in *Pyrrhocoris apterus* (Heteroptera): The relations between life-cycle, chill tolerance and physiological adjustments. J. Insect Physiol..

[74] Käfer H., Kovac H., Simov N., Battisti A., Erregger B., Schmidt A.K.D., Stabentheiner A. (2020). Temperature tolerance and thermal environment of European seed bugs. Insects.

[75] Knapp M., Řeřicha M. (2020). Effects of the winter temperature regime on survival, body mass loss and post-winter starvation resistance in laboratory-reared and field-collected ladybirds. Sci. Rep..

[76] Gouinguene S.P., Turlings T.C.J. (2002). The effect of abiotic factors on induced volatile emessions in corn plants. Plant Physiol..

[77] Loof A.D. (2011). Longevity and aging in insects: Is reproduction costly; cheap; beneficial or irrelevant? A critical evaluation of the “trade-off” concept. J. Insect Physiol..

[78] Cao Y., Li C., Yang W.J., Meng Y.L., Wang L.J., Shang B.Z., Gao Y.L. (2018). Effects of temperature on the development and reproduction of Thrips hawaiiensis (Thysanoptera: Thripidae). J. Econ. Entomol..

[79] Kiritani K. (1988). Eﬀects of climate change on the insect fauna (in Japanese). Meteorol. Res. Rept..

[80] Kiritani K. (1997). The low development threshold temperature and the thermal constant in insects, mites and nematodes in Japan (in Japanese with English summary). Misc. Publ. Natl. Inst. Agro-Environ. Sci..

[81] Kiritani K., Zhang R., Gu D., Zhang W., Zhou C., Pang Y. (1999). Shift of IPM strategy for rice under global warming in temperate areas. Integrated Pest Management in Rice-based Ecosystem.

[82] Ahn J.J., Choi K., Koh S. (2019). Effects of temperature on the development, fecundity, and life table parameters of *Riptortus pedestris* (Hemiptera: Alydidae). Appl. Entomol. Zool..

[83] IPCC (2001). Summary for Policymakers. http://www.ipcc.ch/.IPCC.

[84] Hunter M.D. (2001). Effects of elevated atmospheric carbon dioxide on insect-plant interactions. Agric. Forest Entomol..

[85] Kiritani K. (2006). Predicting impacts of global warming on population dynamics and distribution of arthropods in Japan. Popul. Ecol..

[86] Hong S.Y., Yi H.J., Yoon Y.N., Jang Y.W., Park K.D., Maharjan R. (2022). Evaluation of commercial pheromones on the population dynamics of Spodoptera frugiperda (J.E. Smith) and Mythimna loreyi (Duponchel) (Lepidoptera: Noctuidae). Korean J. Crop Sci..

[87] Maharjan R., Yoon Y., Jang Y., Kim J., Nam H.Y., Jeong M., Park K., Yi H. (2021). Species composition, abundance, and seasonal dynamics of perilla seed bugs (Heteroptera: Lygaeidae) in weeds and perilla fields in Korea. Environ. Entomol..

[88] Honek A. (1999). Constraints on thermal requirements for insect development. Entomol. Sci..

[89] Gilbert N., Gutierrez P.A., Frazer D.B., Jones R.E. (1976). Ecological Relationships.

[90] Jervis M.A., Copland M.J.W., Jervis M., Kidd N. (1996). The Life Cycle. Insect Natural Enemies, Practical Approaches to Their Study and Evaluation.

[91] Haghani M., Fathipour Y., Talebi A.A., Baniameri V. (2007). Thermal requirement and development of Liriomyza sativae (Diptera: Agromyzidae) on cucumber. J. Econ. Entomol..

[92] Young L.J., Young J.J. (1998). Statistical Ecology.

[93] Jianhua M., Stevens M.M. (2021). A degree-day model for predicting adult emergence of the citrus gall wasp, Bruchophagus fellis (Hymenoptera: Eurytomidae), in southern Australia. Crop Prot..

